# Sex differences in vocal communication of freely interacting adult mice depend upon behavioral context

**DOI:** 10.1371/journal.pone.0204527

**Published:** 2018-09-21

**Authors:** Megan R. Warren, Morgan S. Spurrier, Eric D. Roth, Joshua P. Neunuebel

**Affiliations:** Department of Psychological and Brain Sciences, University of Delaware, Newark, Delaware, United States of America; Texas Christian University, UNITED STATES

## Abstract

Ultrasonic vocalizations (USVs) are believed to play a critical role in mouse communication. Although mice produce USVs in multiple contexts, signals emitted in reproductive contexts are typically attributed solely to the male mouse. Only recently has evidence emerged showing that female mice are also vocally active during mixed-sex interactions. Therefore, this study aimed to systematically quantify and compare vocalizations emitted by female and male mice as the animals freely interacted. Using an eight-channel microphone array to determine which mouse emitted specific vocalizations during unrestrained social interaction, we recorded 13 mixed-sex pairs of mice. We report here that females vocalized significantly less often than males during dyadic interactions, with females accounting for approximately one sixth of all emitted signals. Moreover, the acoustic features of female and male signals differed. We found that the bandwidths (i.e., the range of frequencies that a signal spanned) of female-emitted signals were smaller than signals produced by males. When examining how the frequency of each signal changed over time, the slopes of male-emitted signals decreased more rapidly than female signals. Further, we revealed notable differences between male and female vocal signals when the animals were performing the same behaviors. Our study provides evidence that a female mouse does in fact vocalize during interactions with a male and that the acoustic features of female and male vocalizations differ during specific behavioral contexts.

## Introduction

Vocalizations provide a vital means of communication for many species. Animals vocalize in a myriad of contexts, including reproductive behaviors (e.g., [1–3]), aggressive encounters (e.g., [4–6]), and dangerous situations (e.g., [7–9]). In some species, vocalizations are produced by only one of the sexes—typically males (e.g., [10]). In other species, however, all members vocalize, and in these species, there are often differences between male- and female-emitted signals. For example, the pant calls of white rhinoceroses (*Ceratotherium simum*) provide information about age, sex, and social situation [11]. In golden hamsters (*Mesocricetus auratus*), signals emitted by males are of shorter duration than signals emitted by female hamsters [12]. Moreover, individual signals emitted by males typically encompass a smaller range of frequencies than female-emitted signals [12]. Sex differences, however, are not the only factors affecting vocal activity; animals also alter patterns of vocal communication based upon behavioral context. Male tree frogs (*Hyla versicolor*), for example, change the frequency of vocalizations as a function of the intensity of an aggressive encounter, such that lower frequency signals are emitted in more aggressive situations [13]. Gibbons (*Hylobates lar*) modify the features of calls depending on the social context. Calls emitted to indicate the presence of a raptor are shorter in duration and less intense than calls indicating leopards [14]. Taken together, these ideas indicate that both sex and behavioral context play a role in animal communication.

Similar to other species, mice emit vocalizations during social interactions [15]. While mice emit audible vocalizations, the majority of vocalizations produced during social encounters are ultrasonic (USVs) (e.g., [1, 16–20]). USVs are complex [21, 22], contextually modulated [17, 23–25] vocalizations with frequencies exceeding 25 kHz [26]. USVs are emitted at high rates during male-female interactions, but these signals are primarily attributed to the male [27, 28]. Female mice, however, are capable of producing USVs [29, 30]. Therefore, in an effort to investigate sex differences in mouse ultrasonic communication, multiple approaches have been used to distinguish the vocalizations of male and female mice. One approach for examining sex difference in vocal communication is to study mouse pup vocalizations because an isolated male or female pup will vocalize when they are separated from their mother (e.g., [31, 32]). Wright and Brown [32], for example, showed that female pups isolated from their mother produce more vocalizations than male pups. Extending the study of vocal sex differences to adult mice has been more difficult, as adult mice are most vocally active during social interactions, but they show no distinctive visual signature when vocalizing [17]. Thus, determining which animal is vocalizing during social interactions is a challenge and several attempts have been made to overcome this challenge. In 2017, Zala and colleagues [33] allowed males and females to interact through a Plexiglas wall, enabling the determination of which mouse produced specific vocalizations. When males interacted with female mice, significantly more vocalizations were produced than when females interacted with other females. Zala et al., however, did not allow direct physical contact between the animals. Heckman et al. [34] allowed direct nose-to-nose contact by housing mice on two separate platforms and used a sound-source localization system to determine which animal vocalized. The results showed sex differences in the features of vocalizations during semi-restrained interactions between a male and female. Males emitted vocalizations that had lower frequencies and females emitted signals that were longer in duration. In contrast, Hammerschmidt et al. [35] used a same-sex resident-intruder paradigm to show that vocalizations from male and female mice were similar. Because of conflicting evidence and the challenge of quantifying vocal differences during unrestrained social interactions, it is necessary to examine how male and female mice use vocalizations as they freely interact.

In 2015, Neunuebel et al. [20] used a microphone array system to localize vocalizations and identify which mouse produced specific signals while animals freely-interacted. The study revealed, for the first time and contrary to previous beliefs that only males are vocally active during unrestrained mixed-sex group interactions, that females also vocalize. While Neunuebel et al. [20] showed that female mice vocalize when males are present, a systematic comparison between vocalizations emitted by males and females as the animals were freely interacting was never conducted. Consequently, we employed an eight-channel microphone array [36] to track vocal behavior of individual mice (*Mus Musculus*) during unrestrained opposite-sex dyadic interactions and directly compared the vocal activity of males and females across specific behavioral contexts.

## Materials and methods

### Subjects

Adult male (n = 10) and female (n = 8) mice (age: 9–22 weeks) of a B6.CAST-Cdh23Ahl+/Kjn (Cast) background were raised in a mouse colony located in the Life Science Research Facility at the University of Delaware. Cast mice were chosen because mice expressing Cdh23 (homozygous and heterozygous animals) are less likely to suffer from early-onset high frequency hearing loss. Founders of the colony were purchased from Jackson Laboratory (Jackson Laboratory; Bar Harbor, ME; stock number: 002756). At three weeks of age, animals were weaned and group-housed by sex (maximum of 4 per cage) in cages containing ALPHA-dri bedding (Animal Specialties and Provisions, LLC; Watertown, TN; ALPHA-dri) and environmental enrichment. After weaning, mice were implanted with a light-activated microtransponder (PharmaSeq, Inc.; Monmouth Jct, NJ; p-Chip injector), which was used to identify co-housed mice. Tail samples were collected and shipped to TransnetYX, Inc. (TransnetYX, Inc.; Cordova, TN) for genotyping. Only mice expressing Cdh23 were used for experiments. A minimum of two weeks prior to the start of the experiment, mice were isolate housed. Mice remained in isolate housing throughout the experiment in an effort to minimize group housing effects on social behavior [37–39]. Mice were maintained on a 12/12 dark-light cycle (lights on at 9p) with *ad libitum* access to food and water. All experiments were conducted during the dark phase of the light cycle in a room adjacent to the mouse colony.

### Experimental setup

To make males visually distinct from females, the fur of each male was bleached with a five-dot pattern [40] using hair dye (Clairol Born Blonde: Born Blonde Hair Color, Maxi; Proctor and Gamble; Cincinatti, OH). If the dye faded over time, mice were repainted once with the same pattern to ensure that males remained visually distinct from females.

Because previous experience with the opposite sex increases USV emission [41], all mice were exposed to a single animal of the opposite sex for 10 minutes the day after males were first marked with hair dye. Each opposite sex exposure session was conducted in a clean cage with no bedding. An experienced observer terminated the session prior to successful copulation or after 10 minutes. The opposite sex exposure session with the shortest duration lasted 8 minutes. Animals designated as opposite sex stimuli were never used in behavioral recordings.

Thirty minutes prior to a potential recording, non-invasive lavage and assessment of vaginal cells was performed. Although mice are less susceptible to pseudopregnancy than rats [42], care was taken to minimize stimulation and avoid pseudopregnancy. Cells were collected by washing with 30 μl of saline, placed on a slide, and stained with crystal violet. Pictures were taken with a camera (World Precision Instruments, Product number: USBCAM50) attached via coupler (World Precision Instruments, Product number: 501381) to a microscope (VWR, Product number: 89404–890). Estrous stage was determined by assessing the proportion of cell types observed [20, 43]. If the majority of cells were cornified squamous epithelial cells that lacked a nucleus, females were considered to be in the estrus phase of the reproductive cycle and a recording was conducted [20]. A second observer, blind to the estrus designation by the first experimenter, confirmed the estrus state of each female. After determining the female’s estrus state, males and females were weighed and then recordings were started.

In each recording session, a female in estrus was paired with a male for 30 minutes. All males were recorded two or fewer times. Females were recorded a maximum of four times, with the average female recorded less than twice. Pairings were randomly selected without replacement such that a female was never recorded with the same male twice. This controlled for any potential vocal differences due to previous experience with a specific animal. If a successful copulation event, which was defined by the male falling over after an extended period of mounting (n = 2), occurred during a recording, the female was removed from any additionally planned recordings to prevent potential confounds that pregnancy or pseudopregnancy may have on vocal expression.

Each recording was conducted in an anechoic chamber while audio and video data were concurrently recorded. Audio data was simultaneously sampled by each microphone in the 8-channel microphone array (microphones from Avisoft-Bioacoustics; Glienicke, Germany; CM16/CMPA40-5V) at 250,000 Hz using equipment from National Instruments (National Instruments; Austin, TX; PXIe-1073, PXIe-6356, BNC-2110) and low-pass filtered at 200 kHz (Krohn-Hite, Brockton, MA; Model 3384). Each microphone was surrounded by a ring of infrared LED lights that was used to determine microphone position. Video data was recorded with a single camera (FLIR; Richmond, BC, Canada; GS3-U3-41C6M-C) using BIAS software (https://bitbucket.org/iorodeo/bias/downloads/) developed by Kristin Branson, Alice Robie, Michael Reiser, and Will Dickson. The camera was triggered externally at 30 Hz with a counter pulse from the PXIe-6356 card. The counter pulse was simultaneously sent to the National Instruments equipment and the camera through a BNC splitter, which facilitated aligning the video and audio data. Custom-written Matlab software (Mathworks; Natick, MA; version 2014b), was used to control all recording devices. All audio and video data were stored on a PC (Hewlett-Packard; Palo Alto, CA; Z620).

Mice interacted in a mesh-walled (McMaster-Carr; Robbinsville, NJ; 9318T25) cage with a frame of extruded aluminum (width = 76.2 cm, length = 76.2 cm, height = 60.96 cm; 8020, Inc.; Columbia City, IN) surrounded by Sonex foam (Pinta Acoustic, Inc.; Minneapolis, MN; VLW-35). Recordings were conducted in the dark, with infrared lights (GANZ; Cary, NC; IR-LT30) above the cage such that the mice were visible to the camera. Prior to recordings, the floor of the arena was covered with approximately 0.5 inches of ALPHA-dri bedding to enhance contrast between the mice and the cage floor. A ruler was placed in the center of the arena and used to convert camera pixels into meters. Two 15 second pre-tests were run before each recording. In the first, the LEDs surrounding each of the microphones were illuminated and used to determine the position of each microphone. In the second, the LEDs were turned off and the overhead infrared lights were illuminated to confirm the focus of the camera. Following the two pre-tests, the ruler was removed and the infrared lights remained illuminated for the duration of each recording. Recordings consisted of a 30-minute interaction between one male and one female, followed by two ten-minute recordings. For the first ten-minute recording, the female was removed and only the male was recorded. For the second recording, the male was removed and the female was reintroduced and recorded for ten minutes. The two ten-minute recordings were used to train our tracking program to differentiate between the two animals.

### Data processing

A data analysis pipeline was set up on the University of Delaware’s Farber computer cluster. This was used to determine the trajectory of each mouse, as well as information about the vocal signal assigned to each mouse.

### Tracking

Automatic tracking of the mice was completed with the Motr tracking program ([40]; http://motr.janelia.org). In each frame of video, Motr fit an ellipse around each recorded mouse, outputting the x and y positions of the centroid, as well as the major axis, minor axis, and heading direction. Motr used the painted back-pattern to differentiate between the male (five dots) and female (unpainted). Tracking output was visually confirmed by manual inspection in Matlab.

### Audio segmentation

As reported previously [24, 36], automatic vocal signal extraction from the multi-channel array was conducted using multi-taper spectral analysis. Audio data were bandpass filtered between 30 and 110 kHz, then time-overlapping segments were Fourier transformed with multiple discrete prolate spheroidal sequences used as windowing functions (K = 5, NW = 3). An F-test [44] was subsequently used to gauge whether each time-frequency point exceeded the threshold for noise (p < 0.05). This procedure was repeated for a series of segment lengths (NFFTs = 64, 128, 256) on each microphone to capture a range of spectral and temporal scales. All eight channels of audio data were then combined into a single spectrogram and convolved with a square box (11 pixels in frequency by 15 in time) to fill in small gaps. Continuous regions with a minimum of 1500 pixels were automatically extracted as individual signals.

Given that discontinuous vocal signals could originate from one or more animals, each continuous signal was extracted as an individual vocal signal as long as no harmonics were present [20, 36]. Harmonics were defined as overlapping signals where the duration of overlap exceeded 90 percent of the duration of the shortest signal, and the frequencies of these overlapping signals were integer multiples of each other. When harmonics were present, information pertaining to the lowest fundamental frequency was used for analyses. For each extracted signal, a frequency contour (a series of data points in time and frequency) was calculated [24]. Following automatic signal extraction, a custom-written Matlab program was used to manually view the data and confirm that the system was only extracting vocal signals.

### Sound source localization

The sound source localization method is detailed in Warren et al. [36]. Briefly, for each vocal signal, the system sequentially omitted the signal from a single microphone in the array, each time computing an estimate of where the sound originated based upon the remaining seven microphones. Each microphone was omitted a single time for each extracted vocal signal, leading to eight individual estimates per vocal signal. The x- and y-coordinates of the eight estimates were averaged to compute an overall estimate of the location of the source. The eight point estimates, in conjunction with the overall source estimate, were subsequently used to calculate a probability density function over the cage, which allowed us to compute the likelihood that the signal originated from every individual point in the cage. Each animal in the arena was assigned the probability density value (D) at the position of their nose, with nose position determined using the output of Motr. These values were used to calculate a mouse probability index (MPI), indicating the probability that the signal was emitted by each animal in the arena, using the following formula:

$${MPI}_{n}=\frac{D_{n}}{\sum_{i=1}^{M}D_{i}}$$

Where n = mouse number and M = the total number of mice. To assign a signal to a mouse, the MPI value for one of the mice needed to exceed 0.95, indicating a 95% likelihood that the mouse emitted the signal.

### Acoustic analysis

**Fig 1:**
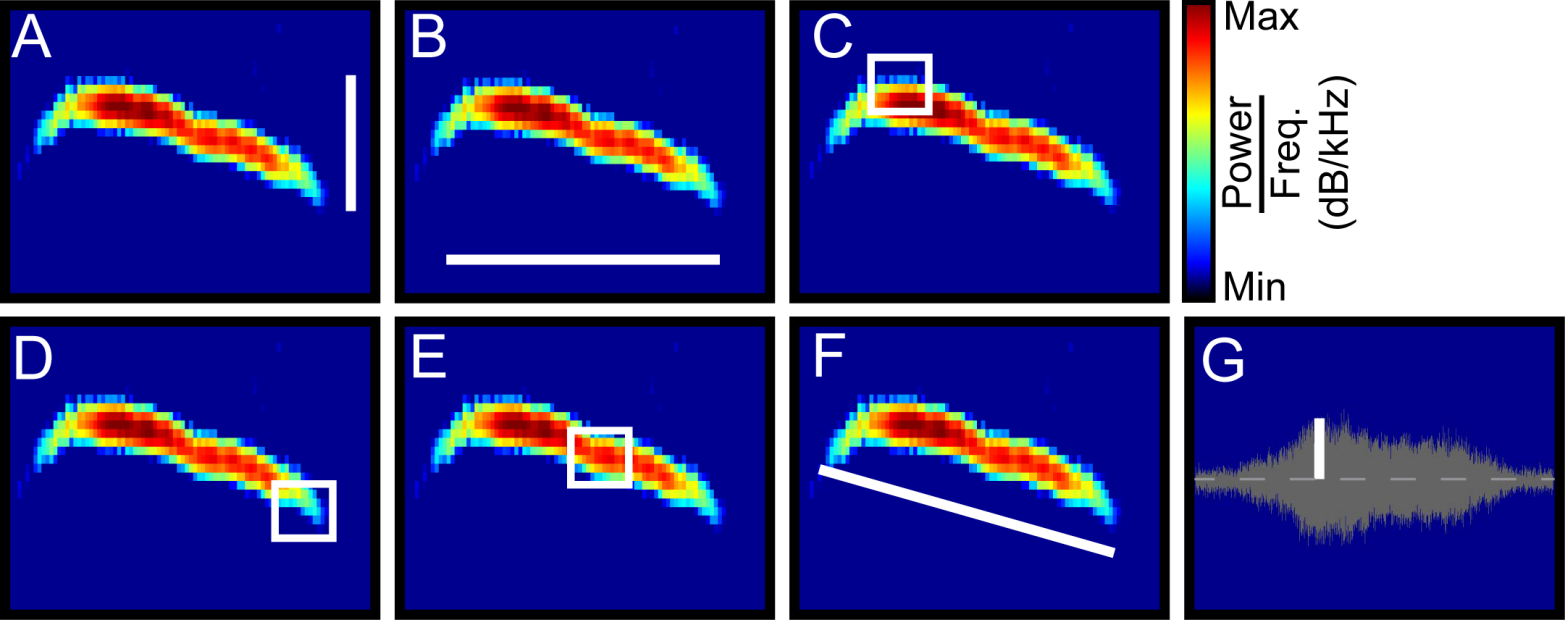
Features of vocal signals. To compare male and female vocal signals, bandwidth (**A**), duration (**B**), high frequency (**C**), low frequency (**D**), mean fundamental frequency (**E**), and change in frequency (**F**) were measured from a single spectrogram. The x- and y-axes represent time and frequency, respectively. The voltage trace (**G**) was used to quantify amplitude, with x- and y- axes representing time and voltage, respectively. The white lines or boxes indicate the quantified features.

To quantify sex differences in vocal signals, a series of vocal parameters were assessed based upon the extracted frequency contours. These included: bandwidth, duration, high frequency, low frequency, mean fundamental frequency, and change in frequency over time (slope). Bandwidth is the total frequency range in a signal (**Fig 1A**), or the highest frequency minus the lowest frequency. Duration of the signal is the difference between end time and start time (**Fig 1B**). High and low frequency are the highest and lowest frequencies in a signal, respectively (**Fig 1C and 1D**). Mean fundamental frequency assesses the average of the power distribution across frequencies (**Fig 1E**). To quantify changes in frequency, a robust fit multilinear regression was applied to the frequency contour of each signal to determine the equation of the line of best fit. The slope of this regression was used to estimate the slope of the vocal signal, defined as change in frequency over change in time (**Fig 1F**). To quantify a signal’s amplitude (**Fig 1G**), a multiple step procedure was followed. First, a voltage trace, corresponding to the start and stop times of the signal, was extracted from the audio data on the first channel of the microphone array. This was repeated for each of the 7 other microphones. Next, an individual sine wave was fit to each of the traces, and the difference between the peak and midpoint of each wave was calculated [45]. This produced 8 amplitude values and the largest value was used as the amplitude of the signal.

### Individual animal contribution

To examine differences between sexes for each vocal feature while controlling for sample size, features were analyzed individually for each mouse. For every vocal feature, the median value was calculated for each individual animal across all recording sessions. Median was used as the measure of central tendency because the distributions of vocal features were highly non-Gaussian as measured by Kolmogorov-Smirnov tests.

### Vocal emission during behaviors

To directly compare male and female signals in the same behavioral context, vocal signals emitted while mice engaged in specific behaviors were examined. Three behavioral contexts were examined: when an animal was not close to the other animal (not close), when a mouse was being followed by another mouse (followed), or when a mouse was following another mouse (following). Four criteria were used to classify following and followed: (1) the positions of the animals relative to each other [front and behind], (2) the direction that the animals were moving relative to each other [separated by less than 25 degrees], (3) the distance between the animals [< 5 cm], and (4) the speed that each animal moved [both > 20 cm/s]. All criteria needed to be met for 10 or more consecutive frames. Mice were considered not close when the centers of the male and female bodies were separated by at least 15 centimeters for 10 or more consecutive frames.

### Statistics

Because data were non-parametrically distributed, as determined by visual inspection and Kolmogorov-Smirnov tests, all statistics were calculated using Mann-Whitney U-tests. Mann-Whitney U-tests were two-tailed with alpha set at 0.05. Pearson’s correlations were calculated using the corr function in Matlab (Mathworks; Natick, MA; version 2016a). All data used for each statistical analysis are available in supporting information (S1 Table).

### Random sampling procedure

To control for differences in the number of vocal signals emitted by sex, a Monte Carlo simulation was conducted to randomly sample the data. For each vocal feature, 1000 separate samples of 500 male-emitted signals and 500 female-emitted signals were randomly selected. The vocal feature of interest was extracted from each of the randomly selected signals and the median value for each sex was computed from the sample. An index value was then calculated using the following formula:

$$\frac{male\;median-female\;median}{male\;median+female\;median}$$

If the median male value exceeded the median female value for that sample, the index value would be positive, indicating a male-driven effect. If instead the female value exceeded the male value, the index would be negative, indicating a female-driven effect. The process was repeated for each of the 1000 samples to provide a distribution of 1000 index values.

Because the index calculation was not sensitive to negative values, we added the absolute value of the most negatively sloped signal to the slope of each vocal signal. The transformation shifted the distribution of slopes such that the signal with the most negative slope had a slope value of zero, and all other slopes were increased by an equivalent amount. Adding a constant value to all of the slopes enabled us to use the index formula, as this made all slope values positive while maintaining the absolute slope differences between signals. Slope values were not shifted for other analyses, as we were interested in quantifying the magnitude of slope differences between groups and the direction of the frequency changes within vocal signals.

### Ethics statement

All experiments were conducted at the University of Delaware in strict accordance with the recommendations in the Guide for the Care and Use of Laboratory Animals of the National Institutes of Health. The University of Delaware Animal Care and Use Committee approved all experimental protocols (protocol number: 1275-2017-0).

## Results

### Vocal expression

**Fig 2:**
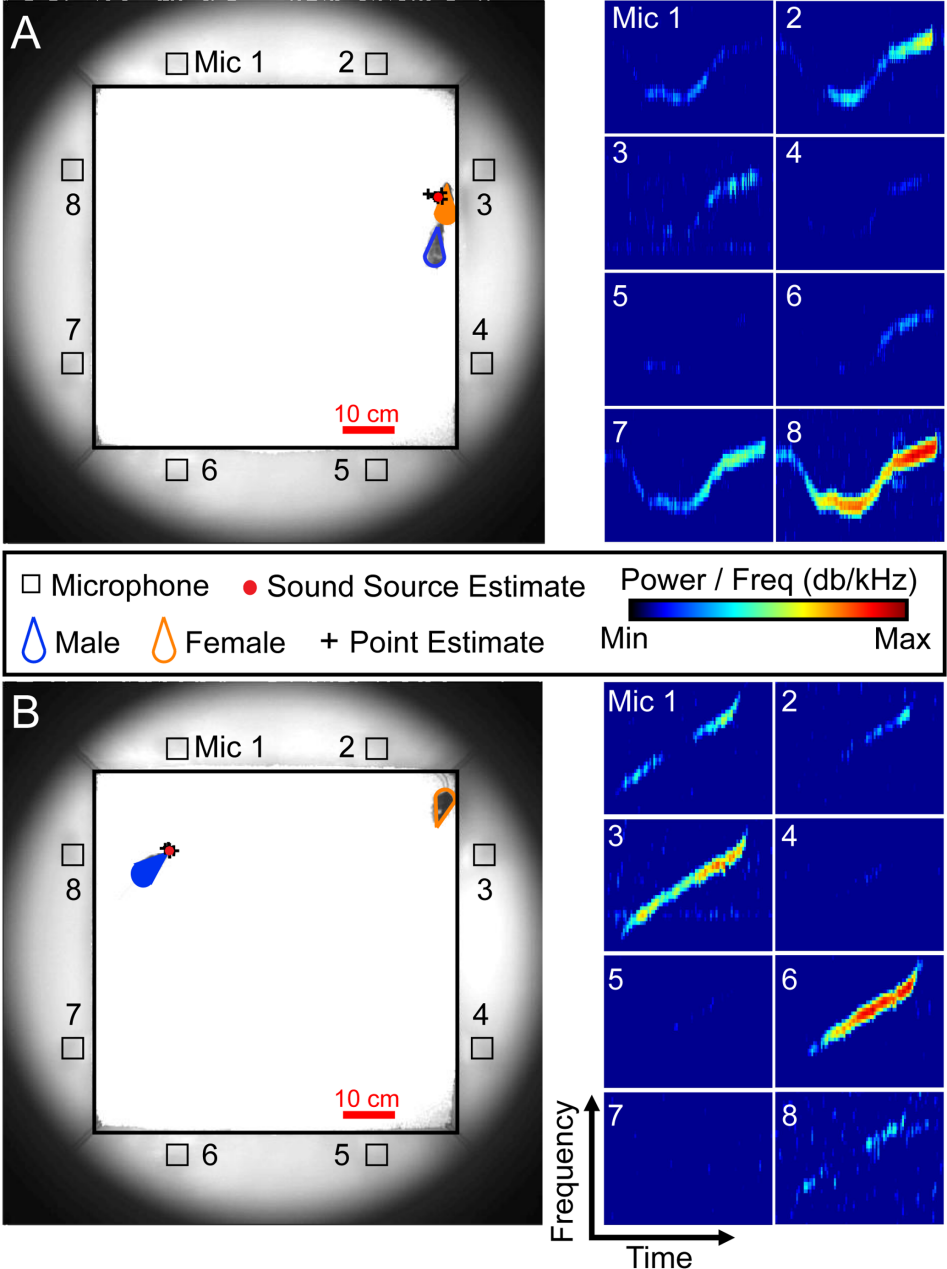
Sound source localization system pinpoints vocal emission during unrestrained dyadic interactions between male and female mice. Examples of signals assigned to either the female (**A**) or the male (**B**). Photograph of the arena (left) at the time of vocal emission indicates the positions of the mice (female = orange, male = blue) and the microphones (Mic 1–8 = □). Filled wedge indicates the mouse that emitted the vocal signal. The vocal signals, which were detected on the microphones surrounding the arena, are shown in the spectrograms to the right.

**Fig 3:**
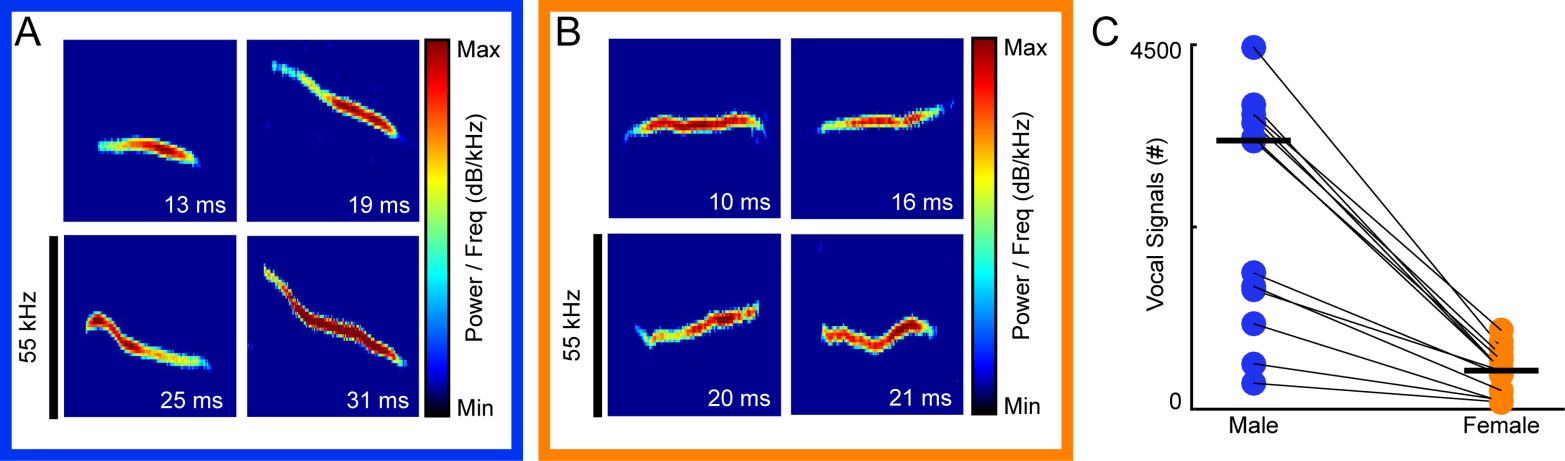
Vocal expression of males and females during dyadic interactions. Representative examples of male (**A**) and female (**B**) vocal signals. X- and y-axis shows frequency and time, respectively. **(C)** Each dot is the number of vocal signals emitted by an animal in a single recording session (n = 13), with males in blue and females in orange. Animals from the same recording are connected by a thin black line. The median value from all 13 data sets is indicated by the thick horizontal black line in each column (median male count = 3318, IQR = 1368–3619; median female count = 477, IQR = 202–622; Mann-Whitney U-test, z = 3.64, p < 0.001).

To investigate sex differences in the vocal signals emitted by male and female mice during unrestrained interaction, a single adult male was paired with an unfamiliar adult female in estrus for 30 minutes, during which audio and video data were concurrently recorded (**Fig 2**). Use of an 8-channel microphone array allowed determination of which vocal signals were emitted by each mouse during naturalistic social interactions. A total of 13 individual dyads were recorded. Across all 13 recording sessions, 55,526 total vocal signals were detected (median per recording = 9,549; IQR = 3,901–10,162), with 38,194 total signals assigned to individual mice. Male mice emitted 84.5 percent of the assigned signals (male = 32,290, female = 5,904). **Fig 3** shows examples of signals emitted by males (**Fig 3A**) and females (**Fig 3B**). In every recording session, the male mouse emitted more vocal signals than the female (**Fig 3C;** median male count = 3,318; IQR = 1,368–3,619; median female count = 477; IQR = 202–622; Mann-Whitney U-test, z = 3.64, p < 0.001), indicating a sex difference in male and female vocal expression.

To determine whether differences in vocal activity could be attributed to size differences between animals, we compared the number of vocal signals emitted and the weight of each animal. There was no significant correlation for either males (r = -0.2, p = 0.5) or females (r = -0.3, p = 0.4), implying that the weight of the animals does not account for the differences in vocal activity. These results indicate that male and female mice vocalize at different rates regardless of the size of the animal.

### Vocal features

**Fig 4:**
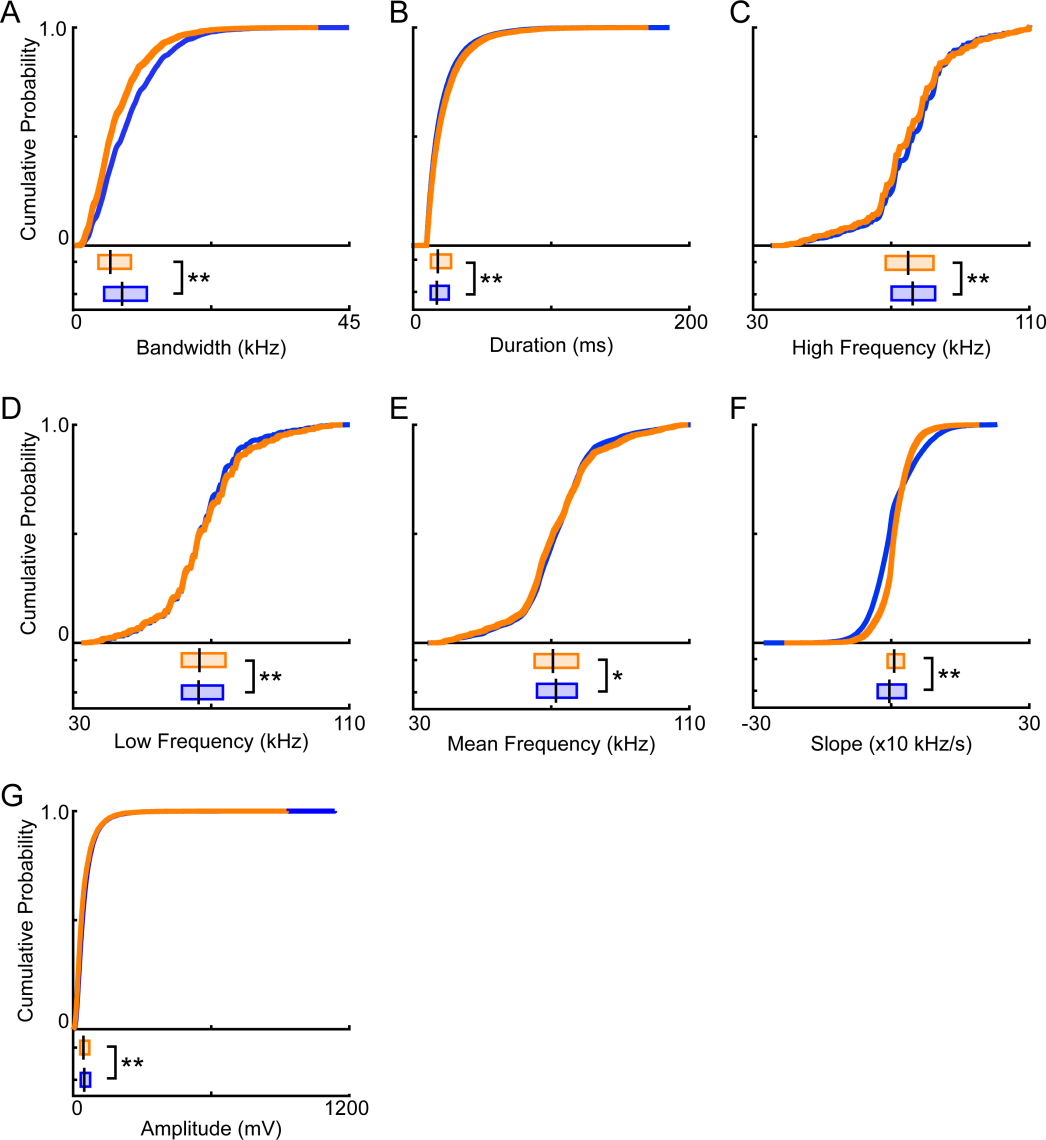
Quantification of sex differences in ultrasonic vocal signals emitted by adult male and female mice. Cumulative density and box plots represent individual vocal features for all signals emitted by either males (blue) or females (orange). Analyses combined data from each recorded dyadic interaction. For box plots, the vertical black line indicates the median value. The left and right edge of each box represents the 25^th^ and 75^th^ percentile, respectively. The vocal features examined were bandwidth (**A**), duration (**B**), high frequency (**C**), low frequency (**D**), mean frequency (**E**), slope (**F**), and amplitude (**G**). Statistical analyses were conducted using Mann-Whitney U-tests. * = p < 0.05, ** = p < 0.01.

While total vocal activity is one metric for measuring sex differences, in other species, male and female vocalizations often differ in other features (e.g. [46–48]). Therefore, to further quantify sex differences in vocal activity in mice, we next examined seven additional features: bandwidth, duration, high frequency, low frequency, mean frequency, change in frequency over time (i.e., slope), and amplitude. Schematics of the previously described vocal features are shown in **Fig 1**. For each of the 38,194 signals assigned to an individual mouse, each feature was measured, then vocal signals were separated by the sex of the emitting mouse. **Fig 4A** shows that male-emitted signals had significantly larger bandwidths than female-emitted signals, with median values of 7.9 kHz and 6.1 kHz respectively (male IQR = 5.1–12.0 kHz; female IQR = 4.1–9.4 kHz; Mann-Whitney U-test, z = 24.6, p <10^−125^). Male signals were, however, of shorter duration than female signals (median male = 17.2 msec, IQR = 12.7–25.9 msec; median female = 17.9 msec, IQR = 12.9–27.5 msec; Mann-Whitney U-test, z = -5.5, p < 10^−6^; **Fig 4B**). The highest frequencies of signals emitted by males, with a median value of 76.2 kHz, significantly surpassed those produced by females, which had a median value of 74.9 kHz (male IQR = 70.1–82.7 kHz; female IQR = 68.4–82.2 kHz; Mann-Whitney U-test, z = 8.0, p < 10^−13^; **Fig 4C**). When comparing the lowest frequencies of emitted signals, male USVs were slightly but significantly lower than female USVs (median male = 66.4 kHz, IQR = 61.5–73.4 kHz; median female = 66.6 kHz, IQR = 61.5–74.1 kHz; Mann-Whitney U-test, z = -3.2, p = 0.0015; **Fig 4D**). The mean frequencies of male-produced vocal signals, however, were marginally higher than those of female produced vocalizations (median male = 71.4 kHz, IQR = 65.9–77.4; median female = 70.5 kHz, IQR = 65.2–77.8 kHz; Mann-Whitney U-test, z = 2.3, p < 0.02; **Fig 4E**). Furthermore, female signals frequently increased in pitch, with a median slope of 0.64x10 kHz/s, whereas male signals frequently decreased in pitch, with a median slope of -0.4x10 kHz/s (male IQR = -2.9x10–3.2x10 kHz/s; female IQR = -0.78–2.8x10 kHz/s; Mann-Whitney U-test, z = -19.1, p < 10^−77^; **Fig 4F**). When comparing the amplitude of signals, male-emitted signals were significantly larger than female-emitted signals (median male = 48.6 mV, IQR = 33.4–74.1 mV; median female = 45.0 mV, IQR = 30.9–70.3 mV; Mann-Whitney U-test, z = 8.1, p < 10^−14^; **Fig 4G**). Thus, all features differed significantly between male- and female-emitted signals.

Although significant sex differences were observed for each acoustic feature, these results may be greatly influenced by sample size, as the probability of making type II errors (i.e., failing to reject the null hypothesis when the null hypothesis is false) is greater with larger differences in sample size. Therefore, to control for the differences observed in the number of vocal signals emitted by males and females, as well as the large sample sizes, we conducted Monte Carlo simulations to randomly sample equal numbers of male and female vocal signals. For each vocal feature examined, we measured the variable of interest from 500 randomly selected male vocal signals and 500 randomly selected female vocal signals. Due to the highly non-Gaussian distributions of each vocal feature, median values were calculated from each random sample. Subsequently, an index value was computed. Briefly, the difference between the male and female medians was calculated and divided by the sum of the two medians. Values above zero indicated that the acoustic feature of interest was greater for male vocal signals, whereas values below zero indicated that the feature was greater for female vocal signals. The process was repeated 1,000 times for each acoustic feature of interest to create a distribution of indices describing the difference between male and female vocal signals. If the distribution of index values was shifted to the right of zero, then the male values for that feature were consistently greater than the female values and the observed sex difference could be attributed to distinct patterns in vocal expression instead of the large discrepancy in sample size. If instead the index values were distributed around zero, with some being positive and some being negative, then the male and female distributions were similar, suggesting that the previously observed sex difference could likely be attributed to differences in sample size between the sexes.

**Fig 5:**
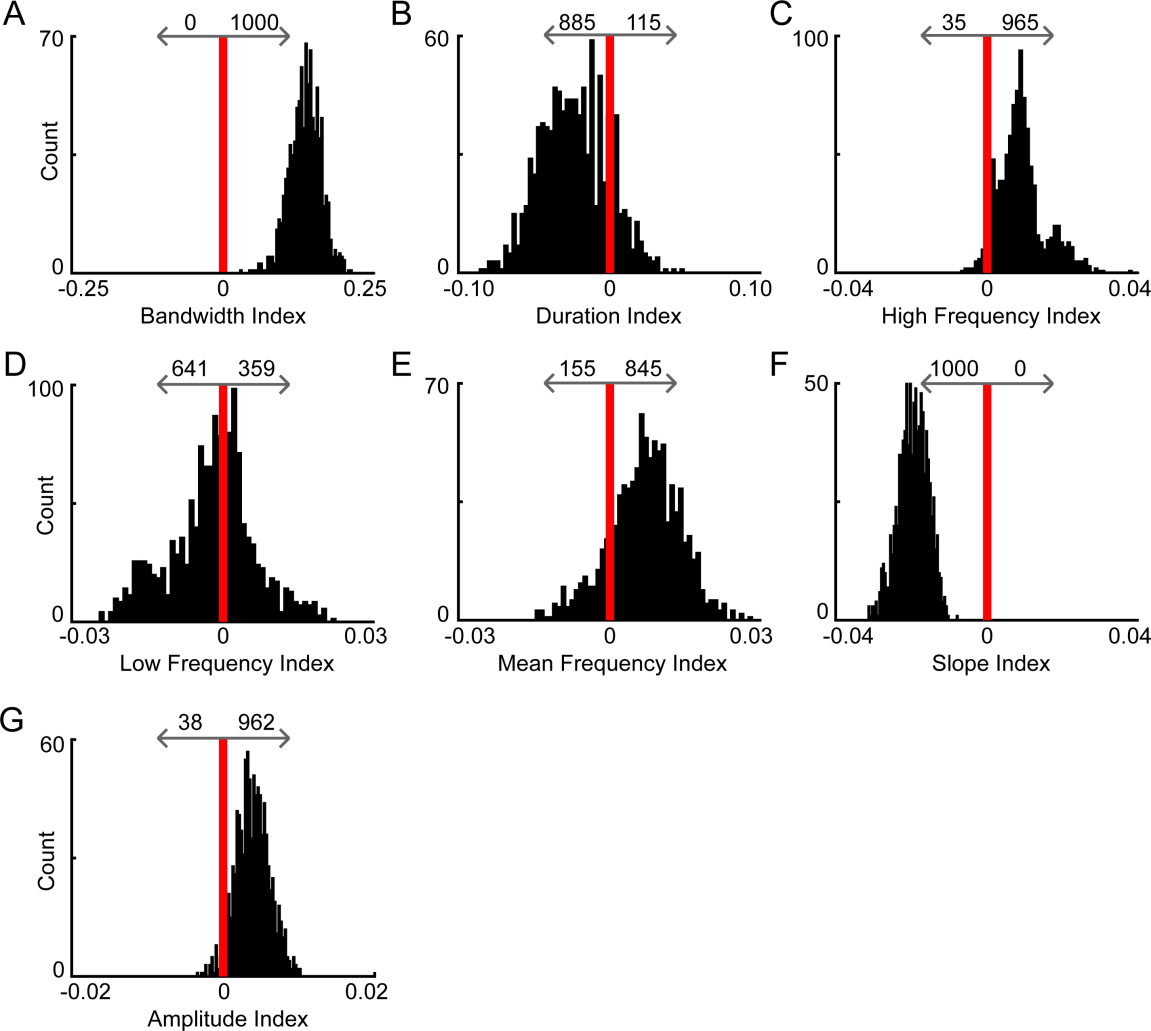
Bootstrap analyses revealed that the bandwidth and slope of vocal signals emitted by male and female mice were consistently different. For each acoustic feature, a subsample of 1000 randomly selected vocal signals (500 male and 500 female) was used to calculate an index quantifying sex differences (see methods). This was repeated 1000 times to create distributions of sex differences for the measured acoustic features. Indices to the right of the vertical red bar (zero line) indicate that the median male value exceeded the median female value from that subsample, while indices to the left indicate the opposite. The total number of indices above zero (male-driven, above arrow pointing towards positive values) and below zero (female-driven, above arrow pointing towards negative values) was calculated. Histograms show bandwidth (**A**), duration (**B**), high frequency (**C**), low frequency (**D**), mean frequency (**E**), slope (**F**), and amplitude (**G**).

**Fig 5** shows that when controlling for sample size, the only sex differences that remained consistently male-driven (males had consistently larger values) or female-driven (females had consistently larger values) were in bandwidth, high frequency, slope, and amplitude. Interestingly, all 1000 index values for bandwidth were greater than zero (**Fig 5A**), indicating that the bandwidths of signals emitted by males were consistently larger than the bandwidth of signals emitted by females. Similarly, high frequency was consistently male-driven (**Fig 5C**), with 96.5% of index values above zero, as was amplitude (**Fig 5G**), with 96.2% of index values above zero. In contrast, all 1000 indices for slope were below zero (**Fig 5F**), suggesting that females emitted signals that increased more rapidly in pitch more often than their male counterparts. There were no consistent trends in duration (885 male-driven, 111 female-driven; **Fig 5B**), low frequency (641 male-driven, 359 female-driven; **Fig 5D**), or mean frequency (155 male-driven, 845 female-driven; **Fig 5E**). Taken together, the results indicate that while significant differences were seen with the previous analysis (**Fig 4**), the differences are not always consistent and may be driven by differences in sample size between groups.

### Individual contribution

The vocal activity of individual mice is highly variable; therefore, differences found between sexes may be driven by a subset of the recorded animals. To control for this possibility, we treated each animal in a recording as a single data point. For each recording, the median value of each vocal feature was calculated across all vocal signals assigned to the male, and the median was separately computed across all signals assigned to the female. Therefore, the data were combined to generate 13 male data points (the 13 median male values) and 13 female data points, allowing us to control for both differences in vocal activity between males and females and inter-individual variability.

**Fig 6:**
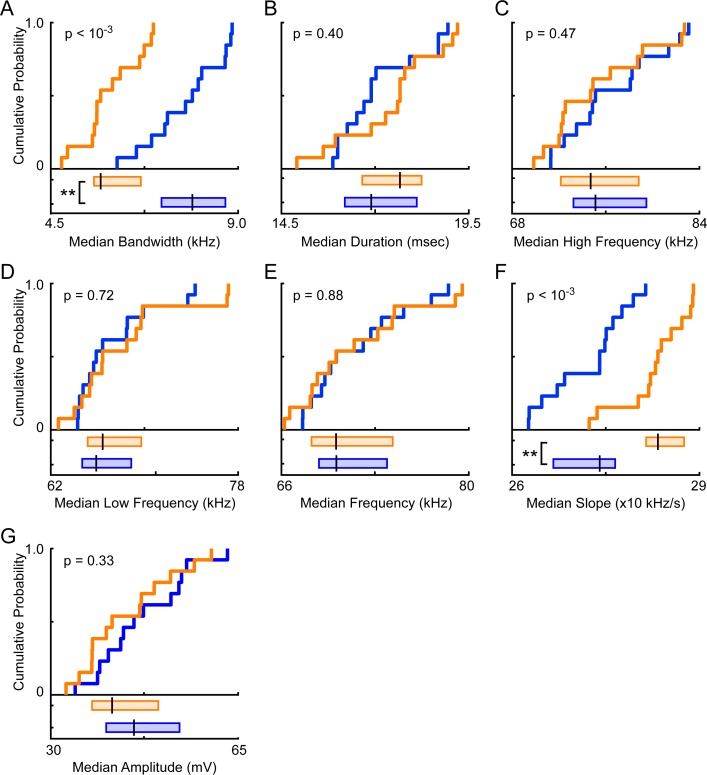
Assessing differences in vocal signals emitted by individual males and females. The median value of each vocal feature for each animal was calculated to directly compare the vocal signalling of males (n = 13; blue) to females (n = 13; orange). Cumulative density and box plots for bandwidth (**A**), duration (**B**), high frequency (**C**), low frequency (**D**), mean frequency (**E**), slope (**F**), and amplitude (**G**). For box plots, the vertical black line indicates the median value. The left and right edge of each box represents the 25^th^ and 75^th^ percentile, respectively. Statistical analyses were conducted using Mann-Whitney U-tests. ** = p < 0.01.

When controlling for the contribution of individual animals (**Fig 6**), only two vocal features remained significantly different between the sexes: bandwidth and slope. Males emitted signals with significantly larger bandwidths than female-emitted signals (median male = 7.9 kHz, IQR = 7.2–8.7 kHz; median female = 5.7 kHz, IQR = 5.5–6.7 kHz; Mann-Whitney U-Test; z = 3.8, p < 10^−3^; **Fig 6A**). No significant difference was detected in signal duration, with a median duration of 16.9 msec for the males and 17.7 msec for the females (male IQR = 16.2–18.1 msec; female IQR = 16.7–18.2 msec; Mann-Whitney U-Test; z = -0.9, p = 0.4; **Fig 6B**). Neither high frequency (median male = 75.1 kHz, IQR = 73.2–79.5 kHz; median female = 74.7 kHz, IQR = 72.2–78.8 kHz; Mann-Whitney U-Test; z = 0.7, p = 0.5; **Fig 6C**), nor low frequency (median male = 65.9 kHz, IQR = 64.7–68.9 kHz; median female = 66.4 kHz, IQR = 65.2–69.7 kHz; Mann-Whitney U-Test; z = -0.4, p = 0.7; **Fig 6D**) differed by sex. Mean frequencies were indistinguishable across sex, with both the male and female median values equaling 70.1 kHz (male IQR = 68.9–73.9 kHz; female IQR = 68.3–74.3 kHz; Mann-Whitney U-Test; z = 0.2, p = 0.9; **Fig 6E**). Slope, however, remained significantly different across sexes, with the average female signal having a significantly more positive slope than the average male signal (median male = 2.7x10 kHz/s, IQR = 2.7x10–2.8x10 kHz/s; median female = 2.8x10 kHz/s, IQR = 2.8x10–2.9x10 kHz/s; Mann-Whitney U-Test; z = -3.4, p < 10^−3^; **Fig 6F**). Amplitude, in contrast, did not differ across sexes (**Fig 6G**). Males emitted signals with an average amplitude of 45.5 mV in comparison to an average of 41.4 mV from females (male IQR = 40.3–54.0 mV; female IQR = 37.7–50.1 mV; Mann-Whitney U-Test; z = 1.0, p = 0.3). These results indicate that, while controlling for the role of individual differences in vocal emission, only two features remain significantly different across sex: bandwidth and slope.

### Vocalizations during behavior

One potential explanation for the differences between male and female vocalizations is that the vocal differences are driven by behavioral context, as the behavioral repertoires of male and female mice are vastly different [49]. As our experimental setup gave us the unique ability to assess vocal activity within a range of unrestricted behaviors, we were able to evaluate the role that behavioral context played on sex differences in vocal communication. Our analyses focused on behaviors in which either animal could participate. For example, both males and females follow members of the opposite sex, whereas mounting, a copulatory behavior, is predominantly performed by males. Therefore, three behavioral contexts were considered. The first behavioral context, not close, occurred when the animals were separated by at least 15 cm. The second behavioral context, followed, occurred when one animal was being followed by the other animal. The third behavioral context, following, occurred when one animal was following the other animal. **Table 1** indicates the number of individual behavioral instances during which at least one vocal signal was emitted and the number of vocalizations emitted during the behaviors.

**Table 1:** Specific behavioral contexts in which vocal signals were emitted.

	Male	Female
**Not close**	637 (5013)*	**48 (51)**
**Followed**	**48 (137)**	**442 (859)**
**Following**	**713 (3386)**	**12 (16)**

*The first number indicates the total number of times that the behavior occurred and the number inside the parentheses shows the total number of vocal signals emitted across all instances of the behavior.

**Fig 7:**
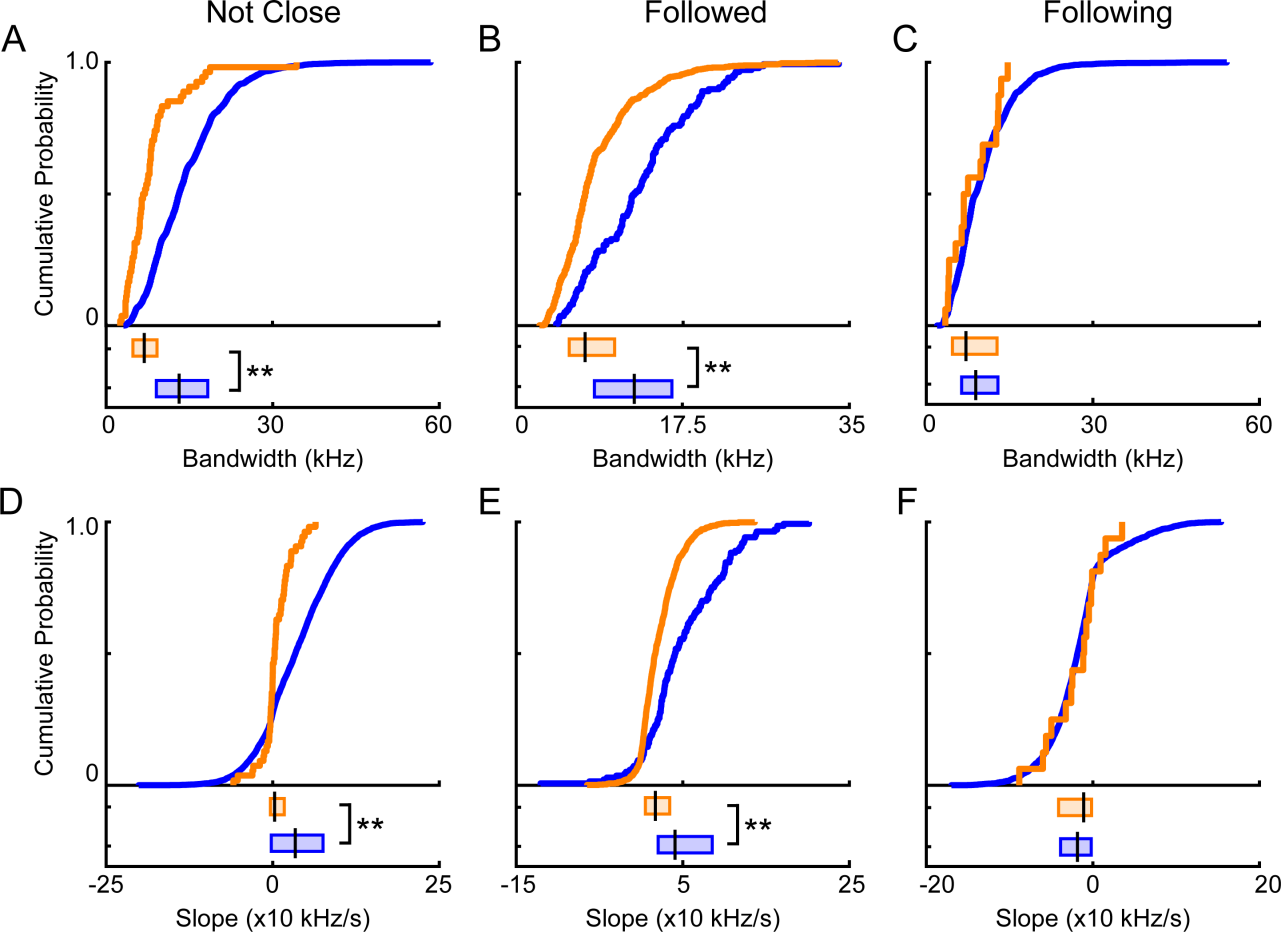
Evaluating differences in vocal signals emitted by males and females in specific contexts. Cumulative density and box plots denote signals emitted by males (blue) or females (orange) in distinct behavioral contexts. Bandwidths of vocal signals emitted when animals were not close to each other (**A**), an animal was in front of another animal (**B**), or an animal was behind another animal (**C**). Slopes of signals emitted when animals were not close to each other (**D**), an animal was in front of another animal (**E**), or an animal was behind another animal (**F**). Statistical analyses were conducted using Mann-Whitney U-tests. ** = p < 0.01.

To determine the extent that behavioral context was the underlying cause of acoustic sex differences, male and female signals were compared within the three behavioral contexts. Because bandwidth and slope were the only features that significantly differed when controlling for sample size and individual variability, we analyzed these acoustic features. The first row of **Fig 7** shows context-dependent differences in the bandwidth of emitted signals. As shown in **Fig 7A**, when animals were not close together, males emitted signals with significantly larger bandwidths than females (male median = 10.9 kHz, IQR = 7.5–15.2 kHz; female median = 5.7 kHz, IQR = 4.0–7.6 kHz; Mann-Whitney U-Test, z = -7.3, p < 10^−11^). For the vocal signals emitted when one animal was being followed by another animal, bandwidths were significantly larger for males than females (**Fig 7B**; male: median = 12.4 kHz, IQR = 8.2–16.6 kHz; female: median = 7.2 kHz, IQR = 5.6–10.3 kHz; Mann-Whitney U-Test; z = -9.6, p < 10^−18^). When one animal was following another animal, however, the bandwidths of male and female vocal signals were similar, with median values of 8.9 and 7.1 kHz respectively (**Fig 7C**; male IQR = 6.3–12.9 kHz; female IQR = 4.7–12.7 kHz, Mann-Whitney U-Test; z = -1.1, p = 0.3).

The second row of **Fig 7** shows context-dependent differences in the slope of emitted signals. When the animals were not close together, the slopes of male emitted signals, with a median value of 3.4x10 kHz/s, were significantly more positive than those emitted by females, with a median value of 0.3x10 kHz/s (**Fig 7D**; male IQR = -0.2–7.5x10 Hz/s; female IQR = -0.4–1.7 x10kHz/s; Mann-Whitney U-Test, z = -4.2, p < 10^−3^). When an animal was followed by another animal, males emitted signals with significantly more positive slopes than their female counterparts (**Fig 7E**; male: median = 4.1x10 kHz/s IQR = 2.0x10–8.5x10 kHz/s; female: median = 1.7x10 kHz/s, IQR = 0.51–3.4x10 kHz/s; Mann-Whitney U-Test, z = -8.3, p < 10^−14^). Notably, a sex difference in the slope of the signals still existed, yet the directionality of the difference flipped compared to when all signals were pooled together. In contrast, when a mouse vocalized while following another mouse, the slope of the signals emitted by males and females was comparable (**Fig 7F**; male: median = -1.9x10 kHz/s, IQR = -4.0x10 - -0.2x10 kHz/s; female: median = -1.1x10 kHz/s, IQR = -4.1x10 - -0.2x10 kHz/s; Mann-Whitney U-Test; z = 0.1, p = 1.0). Therefore, the findings suggest that the sex differences observed in vocal features between males and females may result in part from differences in behavioral repertoire.

**Fig 3** shows a bimodal distribution in the number of vocal signals emitted by males. Some males vocalize more frequently (high vocalizers) and other males vocalize less frequently (low vocalizers). Moreover, there was a significant correlation between the number of signals emitted by males and females that were paired together (r = 0.9, p = 0.0003). Therefore, the sex differences we observed during specific behaviors may be impacted by the differences in vocalization rates. To test this, we split the mice into four groups: (1) a high-vocalizing male group, which emitted more than 2200 signals; (2) a high-vocalizing female group, which consisted of the females paired with the high-vocalizing males; (3) a low-vocalizing male group, which emitted fewer than 2200 signals; and (4) a low-vocalizing female group, which consisted of females paired with the low-vocalizing males.

To determine whether the context-dependent differences in vocal features were impacted by the vocalization rate of the animals, we compared signals emitted by high-vocalizer males to signals emitted by high-vocalizer females, as well as signals emitted by low-vocalizer males to signals emitted by low-vocalizer females. Separating the data into these four groups and comparing the bandwidths of high vocalizers to each other and the bandwidths of low vocalizers to each other produced similar results to the previous analyses (**Fig 7A–7C**). When comparing the bandwidth of signals emitted when animals were not close, we found significant differences between both high-vocalizing males and females (male: median = 11.0 kHz, IQR = 7.5–15.3 kHz; female: median = 6.3 kHz, IQR = 4.1–7.8 kHz; Mann-Whitney U-Test; z = 4.1, p < 10^−3^). The bandwidth of signals produced by low-vocalizing males and females was also substantially different (male: median = 10.7 kHz, IQR = 7.5–14.7 kHz; female: median = 4.9 kHz, IQR = 2.9–5.3 kHz; Mann-Whitney U-Test; z = 2.0, p = 0.04). When being followed, there were significant sex differences in both high-vocalizing (male: median = 12.1 kHz, IQR = 8.3–15.8 kHz; female: median = 7.2 kHz, IQR = 5.6–10.5 kHz; Mann-Whitney U-Test; z = 8.97, p < 10^−17^) and low-vocalizing (male: median = 16.3 kHz, IQR = 6.4–19.0 kHz; female: median = 6.9 kHz, IQR = 4.9–8.9 kHz; Mann-Whitney U-Test; z = 3.1, p = 0.002) animals. For high-vocalizing animals following one another, there were no differences in bandwidth. The median male bandwidth was 8.9 kHz and the median female bandwidth was 7.5 kHz (male IQR = 6.3–12.8 kHz; female IQR = 5.6–12.8 kHz; Mann-Whitney U-Test; z = 0.6, p = 0.6). For low-vocalizing males and females that were following one another, no differences in bandwidth were observed (male: median = 9.5 kHz, IQR = 6.5–13.5 kHz; female: median = 6.3 kHz, IQR = 3.9–10.7 kHz; Mann-Whitney U-Test; z = 1.41, p = 0.2).

When comparing the slopes of signals emitted from high-vocalizing animals in different conditions as well as the slopes of signals emitted from low-vocalizing animals in different conditions, the results were similar to the analyses that did not account for vocal rate (**Fig 7D–7F**). For high-vocalizing animals, there were significant sex differences in slope when animals were not close (male: median = 0.3x10 kHz/s, IQR = -0.03–0.8x10 kHz/s; female: median = 0.03x10 kHz/s, IQR = -0.04–0.2x10 kHz/s; Mann-Whitney U-Test; z = 6.9, p < 10^−10^). Similarly, there were significant sex differences in slope for the low-vocalizing animals that were not close to each other (male: median = 0.3x10 kHz/s, IQR = -0.06–0.8x10 kHz/s; female: median = 0.1x10 kHz/s, IQR = -0.02–0.2x10 kHz/s; Mann-Whitney U-Test; z = 3.8, p < 10^−3^). When animals were being followed, the slope of high-vocalizing males and females differed (male: median = 0.4x10 kHz/s, IQR = 0.2–0.8x10 kHz/s; female: median = 0.2x10 kHz/s, IQR = 0.05–0.4x10 kHz/s; Mann-Whitney U-Test; z = 7.0, p < 10^−10^). The pattern was also observed for low-vocalizing animals (male: median = 1.0x10 kHz/s, IQR = 0.02–1.3x10 kHz/s; female: median = 0.12x10 kHz/s, IQR = 0.05–0.3x10 kHz/s; Mann-Whitney U-Test; z = 3.49, p < 10^−4^). When animals were following one another, the slopes of the signals from high-vocalizing males and females were similar (male: median = -0.2x10 kHz/s, IQR = -0.4 –-0.02x10 kHz/s; female: median = -0.1x10 kHz/s, IQR = -0.4–0.05x10 kHz/s; Mann-Whitney U-Test; z = -0.5, p = 0.6) as were the slopes of signals from low-vocalizing animals (male: median = -0.2x10 kHz/s, IQR = -0.4 –-0.05x10 kHz/s; female: median = -0.3x10 kHz/s, IQR = -0.5 –-0.02x10 kHz/s; Mann-Whitney U-Test; z = 0.09, p = 0.9). Therefore, these findings suggest that context-dependent sex differences in vocal features are present regardless of an animal’s overall vocal activity.

## Discussion

During unrestrained dyadic male-female social interactions, we found that adult males consistently emitted more vocal signals than their female counterparts (**Fig 3**). Subsequent analyses showed that signals emitted by males and females also differed in specific features (**Fig 4**). In particular, the signals produced by males had larger frequency ranges than female-emitted signals, while female-emitted signals increased in pitch more rapidly than male-emitted signals (**Figs 5 & 6**). These results indicate that, during naturalistic social interactions, not only are males and females both vocally active, they also utilize signals with different acoustic features.

Most of the previous studies exploring the utility of USVs assumed that females were not vocally active during male-female interactions (e.g., [16, 50–54]). Using a sound source localization system, both Neunuebel et al. [20] and Heckman et al. [34] showed that female mice are vocally active when interacting with a male, with males producing the majority of the vocalizations. In the Neunuebel et al. [20] study, female mice produced approximately 18% of the vocalizations when multiple male and female mice interacted. Heckman et al. [34] reported that females produced approximately 16% of the vocalizations during semi-restricted dyadic interactions. The current study corroborates the findings of Heckman and colleagues, confirming that females produce approximately 15.5% of the vocalizations. Now that evidence is accumulating to show that female mice vocalize in the presence of males, an important next step should focus on conducting experiments to examine variables that might influence female vocal behavior. Moreover, future analyses should account for female vocalizations when both sexes are simultaneously recorded.

Accounting for female vocalizations could be accomplished in several ways. First, a sound-source localization system specifically developed to track the vocalizations of each individual animal provides the most effective method. This approach distinctly identifies male and female vocalizations, allowing a direct comparison between male- and female-emitted signals. However, the costs and computational demands for processing data collected on a sound-source localization system are high [36]. If this is a concern, a second approach would be removing female vocalizations from the dataset by devocalizing the females. For this approach, stringent controls should be run to confirm that the surgeries effectively devocalize the females and, equally important, do not impact the innate behavior of the surgically manipulated animal. While devocalizing female mice assures that only males are vocalizing, the drawback of this approach is that it limits our understanding of communication between mice.

When quantifying vocal sex differences in other species, the spectral features of vocalizations are frequently examined (e.g., [46–48]), but these features are less commonly investigated in mouse USVs. Instead, analyses examining sex differences in mouse vocal activity are often restricted to call rate [32, 33]. Occasionally, reports have examined the duration of male- and female-emitted vocalizations. Isolated female pups, for example, produce vocalizations with longer durations than males [55, 56], but adult males emit longer signals than females [34]. Our work showed that in adult mice, males emitted longer signals than their female counterparts (**Fig 4**), but the difference was small and inconsistent (**Fig 5**). Moreover, previous studies in both mouse pups [55] and adult mice [34] showed that females modulate the frequency of individual vocalizations more than males. Our results contradict these studies, as we showed that the bandwidth of male vocal signals was significantly larger than that of females (**Figs 4–6**). In previous studies, however, the average difference between male- and female-emitted signals was less than 5 kHz and differences were described as marginally significant. When Hammerschmidt et al. [35] compared the spectral features of male and female mice, no differences in duration, peak frequency, or slope were found. However, the study focused on vocalizations produced during same-sex interactions, which differed from our study. While comparing spectral features of male and female mouse vocalizations can provide a viable metric for quantifying potential sex differences, the disparate results across studies suggests that additional factors should be considered when attempting to interpret sex differences.

Our results indicate that when comparing female-emitted signals to male-emitted signals, two features differ: bandwidth and slope. However, given the importance of context in shaping vocal activity (e.g., [17, 24, 57]), differences in male- and female-emitted signals may be due to the behaviors that the animals engage in as they vocalize. To our knowledge, no previous studies have accounted for the possibility of mouse behavior contributing to sex differences in acoustic communication. To account for behavior, we assessed differences in male- and female-emitted signals within specific behavioral contexts. We found that females emit vocalizations across a range of behavioral contexts, and sex differences in vocal activity were apparent during specific behaviors (**Fig 7**). When animals were not in close proximity, signals emitted by males had significantly larger bandwidths than those emitted by females, and their frequency modulation was also significantly more positive. When an animal was being followed, these differences persisted, with male signals again having significantly greater bandwidths and significantly more positive frequency modulation. However, sex differences in vocalizations were dependent upon behavioral context, as signals emitted when one animal was following the other did not differ by sex. A potential explanation for the lack of sex differences in vocal signals emitted when an animal was following another animal may be differences in sample size, as females did not regularly follow the male. Across all dyads, females emitted only 16 total vocal signals during 12 instances of the behavior (**Table 1**). Males emitted a total of 3,386 vocal signals across 713 instances of the behavior. Thus, it is feasible that sex differences during this behavior may be revealed with a larger sample size. Future work establishing experimental paradigms that increase the number of times that females and males conduct similar behaviors will be essential to further elucidate sex differences.

Both a global analysis of all emitted vocalizations and more a restricted context-dependent analysis revealed sex differences in vocal communication. Interestingly, sex differences in bandwidth were constantly observed across behavioral contexts, with males producing larger bandwidth signals. While sex differences in the slope of vocal signals were evident, our analyses showed that the nature of the differences depended upon context. Grouping all of the signals showed that the slope of the female-emitted signals was more positive than for males. When we instead analyzed signals emitted in specific contexts, female emitted signals were more negatively sloped than male vocalizations. The findings emphasize the importance of investigating signals that are emitted during specific behaviors. When pooling all the vocalizations together, the informational content of the signals may be lost, obscuring potentially important details.

Three possible explanations exist for the differences in male and female vocal signals that we observed during specific behavioral contexts. First, the motivational states of males and females may differ in distinct contexts. During a social interaction, a male may vocalize in an effort to encourage the female to allow him to approach, whereas a female may vocalize to indicate her level of interest in the male. Therefore, when attempting to compare vocalizations between the sexes, specifically assessing behavioral context is essential. A second potential reason underlying differences in male and female signals is inherent biological differences. As can be seen in **Fig 3F**, female signals have a smaller range of slopes than their male counterparts. A potential explanation for this difference is that vocal production in males and females is slightly different, forcing the sexes to rely on vocalizations with different features. Mahrt et al. [58] showed that both male and female mice produce vocalizations by oscillating the superficial vocal folds of the larynx as air is forced through the structure. This indicates that the general mechanism males and females use to produce vocalizations are similar; however, in many species, sex differences in vocal signals are due to other features, such as the size of the animals [59]. Therefore, biological differences between male and female mice may influence the features of the emitted signals. As a third option, there may be a neural basis underlying the sex differences in vocal emission. Recently, Gavrilov and colleagues [60] showed that the primate ventrolateral prefrontal cortex encodes the decision to produce volitional vocalizations. While this has not been tested in mice, Heckman et al. [34] suggested that the neural basis of sex differences in mouse vocalizations may involve differential vocal control by forebrain motor nuclei. Future work directly testing this idea could lead to exciting findings related to the neural basis for sex differences in vocal communication.

## General conclusions

When male and female mice are allowed to interact without restriction and the vocal behavior of each mouse is tracked, notable sex differences in vocal signaling emerge, adding a new level of complexity to the study of mouse communication. During social interaction, further research into vocal activity between animals needs to account for the fact that, not only do both males and females vocalize, but they utilize signals with different acoustic features. By expanding our view to include the perspective that female mice vocalize during interactions with a male, a novel avenue of research opens for further elucidating sex differences between male and female mice, as well as allowing a more in-depth assessment of mouse communication.

## Supporting information

S1 Table Information for each vocalization analyzed. For each vocalization, information is provide indicating the date of the recording, vocalization number, emitter, bandwidth (Hz), duration (ms), high frequency (Hz), low frequency (Hz), mean fundamental frequency (Hz), slope (Hz/s), amplitude, and context.
(XLSX)
